# Convergent Molecular Evolution Associated With Repeated Transitions to Gregarious Larval Behavior in Heliconiini

**DOI:** 10.1093/molbev/msaf179

**Published:** 2025-07-29

**Authors:** Francesco Cicconardi, Callum F McLellan, Alice Seguret, W Owen McMillan, Stephen H Montgomery

**Affiliations:** School of Biological Sciences, University of Bristol, Bristol, UK; School of Biological Sciences, University of Bristol, Bristol, UK; School of Biological Sciences, University of Bristol, Bristol, UK; Smithsonian Tropical Research Institute, Gamboa, Panama; School of Biological Sciences, University of Bristol, Bristol, UK; Smithsonian Tropical Research Institute, Gamboa, Panama

**Keywords:** convergent evolution, molecular evolution, social behavior, gregarious behavior

## Abstract

Collective behavior forms the basis for many antipredator strategies. Within Lepidoptera, larval gregariousness has evolved convergently across many phylogenetically disparate lineages. While the selection pressures shaping variation in larval social behaviors are well investigated, much less is known about the mechanisms that control social attraction and behavioral coordination. Similarly, little is known about how secondary selection pressures associated with social living shape genome evolution. Here, using genomic data for over 60 species from an adaptive radiation of Neotropical butterflies, the Heliconiini, in which gregarious behavior has evolved repeatedly, we explore the molecular basis of repeated convergent shifts toward gregarious larvae. We focus on three main areas of genomic evolution: differential selection on homologous genes, accelerated rates of evolution on noncoding regions of key genes, and differential gene expression in the brains of solitary and gregarious larvae. We identify strong signatures of convergent molecular evolution, on both coding and noncoding loci, in Heliconiini lineages, which evolved gregarious behavior. Molecular convergence is also detected at the transcriptomic level in larval brains, suggesting convergent shifts in gene regulation in neural tissue. Among loci showing strong signals of convergent evolution in gregarious lineages, we identify several strong candidates linked to neural activity, feeding behavior, and immune pathways. Our results suggest social living profoundly changes the selection pressures acting on multiple physiological, immunological, and behavioral traits.

## Introduction

The convergent evolution of similar phenotypes has been a focus of biological research for over a century. Modern sequencing tools have recently allowed the exploration of the genetic bases behind convergent evolution, greatly improving our understanding of the phylogenetic relationships between complex traits such as behaviors (Rosenblum et al. 2014; Sackton and Clark 2019). Molecular changes associated with the evolution of one such behavior, sociality, have received a great deal of attention. Across taxa, individuals living as part of a group are likely to face similar trade-offs, which potentially involve similar molecular functions (Christin et al. 2010; Conte et al. 2012; Fischer et al. 2019; Sackton and Clark 2019). These may include both a molecular response to group living and the molecular control of the behavior itself.

Social behavior can impact a range of selection regimes, which likely shape patterns of genetic divergence and gene expression between solitary and social species. Aggregating introduces stresses like crowding and an increased risk of starvation and pathogen infection (Hochberg 1991; Nilsson and Forsman 2003; Reader and Hochuli 2003; Ezenwa et al. 2016; Li et al. 2021), driving changes in gene expression to mitigate these costs (Wang et al. 2013; Li et al. 2021). Group-living animals may be driven to develop more elaborate neural circuitry to process frequent and potentially complex, social encounters and sensory information in the form of communication signals from group members (Ott and Rogers 2010). Some molecular functions may also be inherently better suited to producing the behavioral phenotype (Christin et al. 2010; Conte et al. 2012; Sackton and Clark 2019), such as those that can be altered without negative pleiotropic effects, or only require slight modifications to produce the new phenotype (Christin et al. 2010; Conte et al. 2012). As such, convergent behavioral evolution may involve similar mechanisms, particularly among close relatives, as the sensory cues available, sensory systems, and downstream neural pathways may have similar biases and variation, and these molecular pathways may require relatively fewer modifications to produce a complex trait such as social behavior.

To examine the extent that convergent behavioral changes involve similar molecular mechanisms, we focus here on larval gregariousness in Lepidoptera. These larvae vary widely in the size, longevity, and nature of their social groups. Even between relatively closely related species, these larvae vary widely in the size, longevity, and nature of their social groups (reviewed in McLellan and Montgomery 2023). Group sizes may range from tens to hundreds of individuals, aggregations may disperse after the first two instars or remain together up to pupation, and group members may simply feed in the same place or and exhibit trail-following and synchronized defensive actions that require group coordination (Costa and Pierce 1997; Hunter 2000; Costa 2006; Greeney et al. 2012). Allied to these behavioral traits is huge diversity in morphology, host plant ecology, life history, and development (Costa and Pierce 1997; Hunter 2000; Costa 2006; Greeney et al. 2012). This diversity positions larval Lepidoptera as an informative window into the convergent evolution of group living. Gregarious behavior in larvae has evolved convergently across many phylogenetically disparate lineages of Lepidoptera (Sillén-Tullberg 1988; Tullberg and Hunter 1996; McLellan et al. 2023). Yet, to date, research into the evolution of social behavior in larval Lepidoptera has focused almost completely on ultimate causes and ecological contexts (Rittschof and Robinson 2014; Despland 2021; Rentería et al. 2022; McLellan and Montgomery 2023), with little consideration for the genetics driving the behavior. This previous work highlights the phenotypic and environmental conditions that are important for gregariousness to evolve (McLellan et al. 2023; McLellan and Montgomery 2023, 2024), yet almost nothing is known of the genomic alterations required to produce the behavior, or the effect that the behavior-induced selection regimes have on the genome.

Here, we focus on the Heliconiini, a diverse tribe of Neotropical butterflies across which larval gregariousness has evolved independently on at least seven separate occasions (Beltran et al. 2007; McLellan and Montgomery 2024), as a potentially powerful system in which to investigate the molecular basis of social behavior. Using genomic data for 53 species in the Heliconiini tribe and additional closely related taxa (Cicconardi et al. 2023), we conduct the first phylogeny-wide study testing whether convergence in social behaviors is associated by a molecular convergence and shared selection regimes. We examine shifts in selection pressure on orthologous genes across Heliconiini lineages with gregarious and solitary larvae, as well as testing for accelerated rates of evolution in noncoding regions associated with key loci. Finally, we compare gene expression profiles of three Heliconiini species with gregarious larvae, from three separate subgenera monophyletic groups, here defined as clades, in relation to three related species with solitary larvae, creating three monophyletic pairs of social and solitary species.

## Materials and Methods

### Detecting Signatures of Selection and Convergence Associated With the Gregarious Phenotype

To detect a signal of association between the gregarious phenotype with a shift in selective pressure (d*N*/d*S* or *ω*) across protein-coding genes (PCGs), we used the 3,393 single-copy orthologous groups (scOG) list across the Heliconiinae phylogeny from Cicconardi et al. (2023) and a set of computational approaches designed to explore the selective landscape operating across the genome. These methods detected either general shifts in selection between all gregarious and solitary lineages or between specific lineages that have independently evolved gregarious larvae. We treat both as evidence of convergent molecular evolution.

The first method, Branch-site Unrestricted Statistical Test for Episodic Diversification for PHenotype (Busted-ph), is a method to test for evidence of episodic diversifying selection associated with a specific feature/phenotype/trait (Kosakovsky Pond et al. 2020; Pond 2023). The method first tests whether both background branches and test branches experience episodic positive selection then checks if there is a difference between the two partitioned *ω*. We specifically tested whether there is a difference between lineages associated with the gregarious phenotype versus all solitary lineages. The resulting *P*-values were corrected for multiple testing with the Benjamini–Hochberg method (“BH” FD“FDR”) as implemented in the R package P.adjust {stats}, using an alpha threshold of 0.05. Because the within group test of episodic selection gives more stratified results rather than just differentially selected or not, we divided scOGs into two groups: (i) *associated with gregariousness* if there is a difference of selection between gregarious and nongregarious (adjusted *P* < 0.05), but not if episodic positive selection occurred in both gregarious and nongregarious (*P* < 0.01), and (ii) *not associated with gregariousness* for the other cases. In other words, we exclude genes under selection in both gregarious and nongregarious lineages. The two groups were plotted comparing their reciprocal gregarious *ω* versus solitary *ω*, and their relative scaling coefficients and intercepts were analyzed with SMATR (Warton et al. 2012).

We also tested whether scOGs experienced relaxation or intensification by comparing gregarious versus solitary lineages with Relax (Wertheim et al. 2015), as implemented in the HYPHY batch language (Kosakovsky Pond et al. 2020). More specifically, Relax tests whether selection pushes all *ω* categories away from neutrality, i.e. intensification (1 > *ω* > 1), or toward neutrality, i.e. relaxation (*ω* = 1). A *k* parameter is computed, which is lower than 1 (*k* < 1) in cases where the model supports the relaxed scenario or higher (*k* > 1) where intensification is better supported. Also here, *P*-values were corrected for multiple testing with the Benjamini–Hochberg method, and adjusted *P*-values < 0.05 were considered as significant.

Finally, we used Csubst (Fukushima and Pollock 2023) to derive observed nonsynonymous convergence (*O_C_^N^*) and observed synonymous convergence (*O_C_^S^*), expected nonsynonymous convergence (*E_C_^N^*), and nonsynonymous convergence (*E_C_^S^*) to evaluate their rates (*dN_C_* and *dS_C_*) and the relative *ω_C_* metric ((*O_C_^N^*/*E_C_^N^*)/(*O_C_^S^*/*E_C_^S^*)), in combinations of branches in phylogenetic gene trees. We ran the analysis twice, once accepting all convergent events that occurred only in gregarious lineages with a threshold of *ω_C_* > 1.0 and *dN_C_* (*O_C_^N^*/*E_C_^N^*) > 1.0, as an overall signal of convergence, and again with a more stringent threshold of *ω_C_* > 5.0 and *dN_C_* > 2.0 for an *arity* ≥ 2 (degree of combinatorial substitutions or the number of branches to be compared, or number of independent branches in a phylogenetic tree) to be considered convergence. Other statistical tests such as the one-sided Wilcoxon rank-sum tests were used to test for differences in the distributions and the Fisher exact test to examine whether the overlap between gene sets deviates from a random distribution. Both statistical tests were run as implemented in R and Scipy.stats.

### Annotating CNEEs

To identify conserved nonexonic elements (CNEEs), we used the 63-way whole-genome alignment generated by Cicconardi et al. (2023), together with the model of neutral evolution computed with Phast v1.4 package (Siepel et al. 2005; Hubisz et al. 2011). Because the majority of gregarious lineages belong to the genus *Heliconius*, we recomputed the conserved and accelerated models using Phastcons with the *Heliconius melpomene* genome as reference, combining the conserved and nonconserved models with Phyloboots and using the averaged models to predict conserved elements in Phastcons, using default parameters. After the initial estimation of conserved elements, we merged regions within 5 bp of each other into a single conserved element. We then excluded regions (i) shorter than 50 bp or (ii) with data for <50 species for that region and (iii) with more than 50% of gaps in the consensus sequence, following the protocol used in Cicconardi et al. (2023). We used PhyloAcc-GT (Yan et al. 2023), which computes the maximum *a posteriori* Z matrix (matrix of latent states), and two Bayes factors to test for acceleration at the gregarious stems: a Bayes Factor 1, defined as the Bayes factor that compares a null model (no acceleration allowed on any branch) to the test branch model (acceleration allowed only on gregarious branches) (M1), and a Bayes Factor 2 defined as the Bayes factor comparing the test model to the full model (acceleration allowed on any branch) (M2). From all accelerated CNEEs (aCNEEs) under the M1 model, we defined as “*strict*” those which affect at least three different monophyletic lineages that evolved the gregarious behavior, also defined here as clades (e.g.: Doris, Sara/Sapho, and *Dione juno*). We also used PhyloP from Phast v1.4 package (Siepel et al. 2005; Hubisz et al. 2011) [--mode CONACC --wig-scores --method LRT] to generate the acceleration/conservation scores.

### Enrichment Among CNEEs

To test for gene ontology (GO) terms of functional elements enriched in gregarious aCNEEs, we used two approaches implemented in Cicconardi et al. (2023): (i) two permutation approaches to account for possible biases toward particular gene functions and (ii) a genomic fraction approach as implemented in Great (McLean et al. 2010). To each gene in the reference genome annotation (*H. melpomene*), a regulatory domain was assigned as the 5 + 1 kb with extension strategy, similarly to Great (McLean et al. 2010). In brief, to each coding locus, a regulatory domain is assigned consisting of a basal domain that extends 5-kb upstream and 1-kb downstream from its transcription starting site (TSS), which may overlap between other basal domains of flanking loci, plus a further extension upstream to the basal domain, up to 1 Mb. This 1 Mb is a soft extension as it cannot overlap the basal domain of another gene. It is implemented to incorporate gene deserts that are rich in regulatory regions, but will not impact gene-dense regions, which are the majority of our insects’ genomes.

The “simple permutation test” was computed calculating the expected probability of overlap between the regulatory domains of genes of a specific GO term and 10,000 random aCNEEs datasets, shuffling the CNEEs and randomly selecting the same amount of aCNEEs among all CNEEs, using a binomial test to generate a *P*-value. For the second test, “genomic region test,” similarly to Great, the binomial test was executed over the total fraction of genomic regions associated with a given GO term. A third method was implemented “reshuffling permutation set” in which Bedtools shuffle [-excl gff3 -chrom -chromFirst -noOverlapping] was adopted to shuffle all detected aCNEEs across the entire genome avoiding overlaps with annotated genes and comparing the overlap with putative regulatory elements, in a binomial test framework. Multiple test correction was performed with the Benjamini–Hochberg Fdr (Fdr_bh) and Bonferroni as implemented in Python library Statsmodels.Stats.Multitest. With both approaches, we tested all biological processes where at least two aCNEEs were overlapping with a regulatory domain of the given GO term. We used REVIGO (Supek et al. 2011) to summarize results.

In the same fashion, we also looked for genomic regions, gene annotations, and gene sets (Busted-ph) enriched for aCNEEs. To do this, we generated nonoverlapping 100-kb sliding windows and computed the probability of observing aCNEEs based on the binomial distribution over the 10,000 permutations of randomly selected aCNEEs among all CNEEs, where the number of trials is the number of aCNEEs in the window and the probability of success is the average of the permutations; the excess of aCNEEs per regulatory domain using the distribution over the 10,000 permutations as implemented in Cicconardi et al. (2023); and the excess of aCNEEs to the gene of differentially selected scOGs (Busted-ph) over 10,000 permutations considering all the genomic regions/regulatory domains of all the tested scOGs as the background. The *P*-values of those tests were then corrected as before, adopting alpha threshold equal to 0.05.

Motif enrichment for strict aCNEEs overlapping with the putative regulatory regions of Busted-ph positive scOGs was done using FindMotifsGenome.pl within HOMER2 suite (Duttke et al. 2024) using all strict aCNEEs overlapping with all tested scOGs.

### Larval Rearing and Sample Collection

Brain tissue from larvae of six species from the Heliconiini tribe was used for the transcriptomic analysis: *D. juno*, *Heliconius doris*, and *Heliconius sara*, which are all gregarious as larvae, and *Agraulis vanillae*, *Heliconius hecale*, and *Heliconius erato*, which are solitary. These six species belong to three distinct monophyletic pairs of gregarious and solitary species and were used to contrast gregarious lineages to solitary close relative species. Although behavioral variation exists among gregarious species, we selected these species for comparison because all three remain gregarious up to, and often including, pupation (Muyshondt and Young 1973; Sobral-Souza et al. 2015; Jiggins 2017). Stock populations of each species were established from wild-caught females collected around Gamboa, Panama, in 2013. All sampled larvae were first- or second-generation, insectary-reared individuals, with the exception of the locally less abundant *D. juno*, which was opportunistically sampled from a single brood found as eggs on a *Passiflora vitifolia* in the outdoor host plant stocks. Stock populations were held in ambient, tropical conditions outside in ∼2 × 2 × 3 m cages were fed on artificial feeders and for *Heliconius* spp. with ∼20% sugar/bee pollen solution with supplemented floral resources (*Lantana* sp. and *Psiguria* sp.). Each species had access to their preferred host plant (*D. juno* and *H. hecale*: *P. vitifolia*; *H. doris*: *Passiflora ambigua*; *H. sara*: *Passiflora auriculata*; *A. vanillae* and *H. erato*: *Passiflora biflora*). Eggs were collected daily, and larvae were subsequently reared on shoots in pop-up cages at ambient conditions. All species were reared concurrently so were exposed to common environmental conditions. Larvae were sampled in the early fifth instar, before the prepupal instar, or “wandering stage,” where behavior often changes drastically as larvae cease foraging and search for a pupation site (see for example Smallegange et al. 2007). We used five separate individual samples per species, giving a total of 30 samples. For the gregarious species, *H. doris* and *H. sara* samples were taken from individuals from four separate broods, whereas all five *D. juno* samples were taken from the same brood. Solitary species were selected from the same generation, but as eggs were derived from stock cages with multiple fertilized females, the kinship between individuals was not known. The brain and connecting subesophageal ganglion were dissected out in *RNAlater* (which stabilizes RNA, preventing its breakdown; Thermo Fisher Scientific), and stored at −20 °C.

### RNA Extraction and Sequencing

We extracted total RNA from each sample consisting of an intact, attached brain, and subesophageal ganglion following the instructions of the RNeasy Micro Kit according to the manufacturer's protocol (Qiagen). RNA concentration, purity, and integrity were assessed on an Agilent 4150 TapeStation (Bristol Genomics Facility, UK). To improve 28S RNA peak visibility, samples were not subjected to heat denaturation treatment, following the suggested approach for assessing insect RNA (Winnebeck et al. 2010). All samples had an RNA Integrity Number of ≥9, indicating that the RNA was of suitable quality for sequencing (Schroeder et al. 2006). Samples were sent to Novogene (UK) for library preparation (with poly A enrichment) and transcriptome sequencing. Libraries were sequenced on an Illumina NovaSeq 6000 platform, producing ∼150-bp paired-end reads. We quality checked raw reads using Fastqc v. 0.11.9 (Andrews 2010) and trimmed them using Trimmomatic v. 0.39 (Bolger et al. 2014) to remove adapter contamination, low-quality reads, and reads shorter than 75 bp, using default command line settings.

### Differential Gene Expression Analysis

Annotated genomes for all six Heliconiini species were obtained from Cicconardi et al. (2023). We used STAR v. 2.7.11a (Dobin et al. 2013) to map trimmed reads to genomes and HTSeq v. 2.0.3 (Anders et al. 2015) to count the mapped reads per gene. A table of Heliconiini orthologous groups (OGs) was obtained from Cicconardi et al. (2023) and filtered to retain only scOGs containing genes for all of our target species (total retained genes = 6,885). To note that the list of scOGs here is larger than the overall scOG list used previously, due to the reduced species list having higher overlap in single-copy genes. Among each pair, there are 8,841 scOGs between *A. v. vanillae* and *D. juno*, 9,200 between *H. doris* and *H. hecale*, and 9,684 between *H. sara* and *H. erato demophoon*. We then matched the read counts of genes to their corresponding OGs to compare OG expression across the study species.

All analyses of differential gene expression were performed in R v. 4.1.2 (R Core Team 2013) using Limma v. 3.5.3 (Phipso et al. 2016). Raw read counts were first normalized based on gene length, library size, and GC content using CQN v. 1.4.0 (Hansen et al. 2012), to account for potential bias due to differences in homologous gene lengths between species (Zhou et al. 2019).

We compared gene expression between species in three separate pairs based on larval social behavior and clade, where each pair consisted of a gregarious and solitary species belonging to the same clade: (i) *D. juno* versus *A. vanillae* (diverged ∼14 mya, (ii) *H. doris* versus *H. hecale* (diverged ∼9 mya), and (iii) *H. sara* versus *H. erato* (diverged ∼5 mya) (Cicconardi et al. 2023). Additionally, we performed a full comparison of all three solitary species versus all three gregarious species. Differentially expressed genes (DEGs) were selected using adjusted *P*-value threshold of 0.05. Of these, we selected only those with a minimum log_2_ fold change (log_2_FC) of ±1 to account for any differences in brain tissue scaling (overall size, region volumes, etc.) between species (Montgomery and Mank 2016). Finally, in order to identify adaptive shifts or convergence across our phylogeny, we implemented phylogenetic multioptima Ornstein–Uhlenbeck (OU) models, that is, Hansen models (Hansen 1997); with the Estimate_shift_configuration and Estimate_convergent regimes functions in the R package L1ou v1.43 (Khabbazian et al. 2016), to detected as multiple regime shifts that lead to similar expression levels, convergent regime shifts (Khabbazian et al. 2016; Fukushima and Pollock 2023).

### Functional Enrichment and Overlap of DEGs

A list of GO terms corresponding to our OGs was obtained from Cicconardi et al. (2023), providing information on the biological function toward which each DEG is likely to contribute (Ashburner et al. 2000). These GO terms were used for functional enrichment analyses, using the R package topGO v. 2.46.0 (Alexa and Rahnenführer 2009) on each species pair, with the aim of highlighting the functions that are overrepresented in our gene lists of interest, both downregulated and upregulated.

Finally, we examined the overlap of DEGs between the three species-pair comparisons to identify convergent shifts in expression. We used 10,000 iteration permutation tests to assess the probability that the number of shared DEGs between the two- and three-way species-pair comparisons is greater than expected by chance. In detail, *n* genes were sampled without replacement from the full list of OGs, where *n* = the number of observed DEGs from a given species pair, to create a list of random DEGs. The number of shared genes between both (or all three) randomized lists was recorded, and the process repeated over 10,000 iterations to give a distribution of 10,000 sets of shared genes. We then tested the observed count of shared DEGs against this distribution for the probability of it occurring by chance.

## Results and Discussion

### Convergent Shifts in Selection Regime Shape Coding Genes in Gregarious Species

**Fig. 1.**
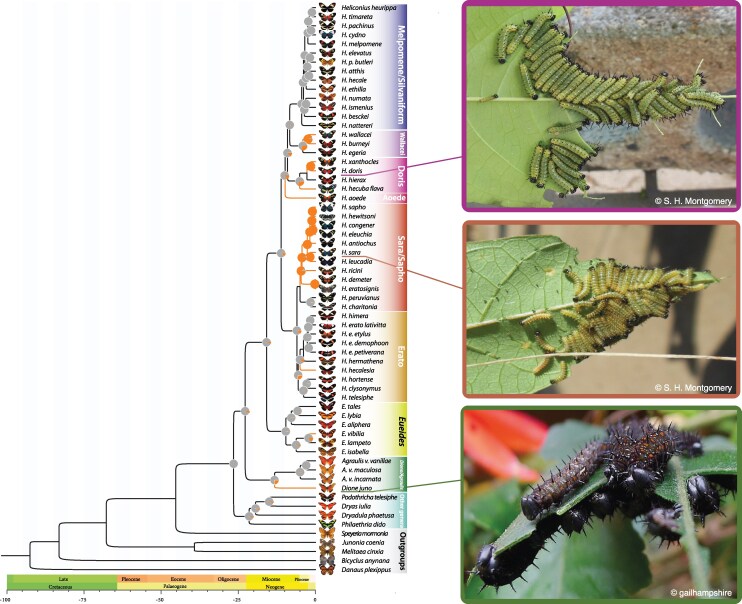
Heliconiini phylogeny and ancestral state reconstruction of gregarious behavior. A dated phylogeny of Heliconiini species from Cicconardi et al. (2023). Orange branches indicate lineages having gregarious behavior, while pie charts indicate the maximum likelihood estimate of the most recent common ancestor to have gregarious larvae from McLellan and Montgomery (2024). Photographs illustrating independently evolved larval social groups in (from top to bottom) *H. doris*, *H. sara*, and *D. juno*. Photograph of *D. juno* by Gail Hampshire, used under CC 2.0, via Wikimedia Commons.

Using a set of scOGs obtained from 53 Heliconiinae species (Fig. 1) (Cicconardi et al. 2023), we first explored the strength of convergent evolution among PCGs between gregarious species. To do so, we adopted three analytical approaches: (i) using Busted-ph (Kosakovsky Pond et al. 2020; Pond 2023), we tested for evidence of different selective regimes between solitary and gregarious lineages; (ii) using Relax (Wertheim et al. 2015), we tested whether these shifts in selection regime can be explained with a relaxed selection pressure (a shift of rate classes toward neutrality; *k* < 1) or with an intensification of selection pressure (a shift of rate classes toward purifying and positive selection; *k* > 1); finally, (iii) we scanned scOGs for signs of convergent substitution with Csubst, a method for detecting the combinatorial SUBSTitutions of codon sequences (Fukushima and Pollock 2023). The Csubst algorithm is designed to detect adaptive molecular convergence, specifically convergent nucleotide substitutions in codons of PCGs. It corrects for false positives due to phylogenetic uncertainty and neutral evolutionary noise. Csubst extends the classic *dN/dS* ratio (*ω*) by computing: *ω_C_*, which is calculated computing the rage of observed/expected nonsynonymous convergent substitutions (*dN_C_*) over the rate of observed/expected synonymous convergent substitutions (*dS_C_*). Because synonymous substitutions are assumed to be neutral, they serve as a baseline for false convergence due to phylogenetic effects. Thus, if *dN_C_* increases due to phylogenetic effects, *dS_C_* tends to rise too, but true adaptive convergence results in an increased *dN_C_* without a corresponding increase in *dS_C_*, which pushes *ω_C_* > 1 (*ω_C_^+^*) (Fukushima and Pollock 2023). Csubst scans all combinations of target lineages from pairwise (*arity* = 2) to multiple (*arity* > 2) comparisons to identify genes that are under convergent positive selection (*ω_C_^+^*).

**Fig. 2.**
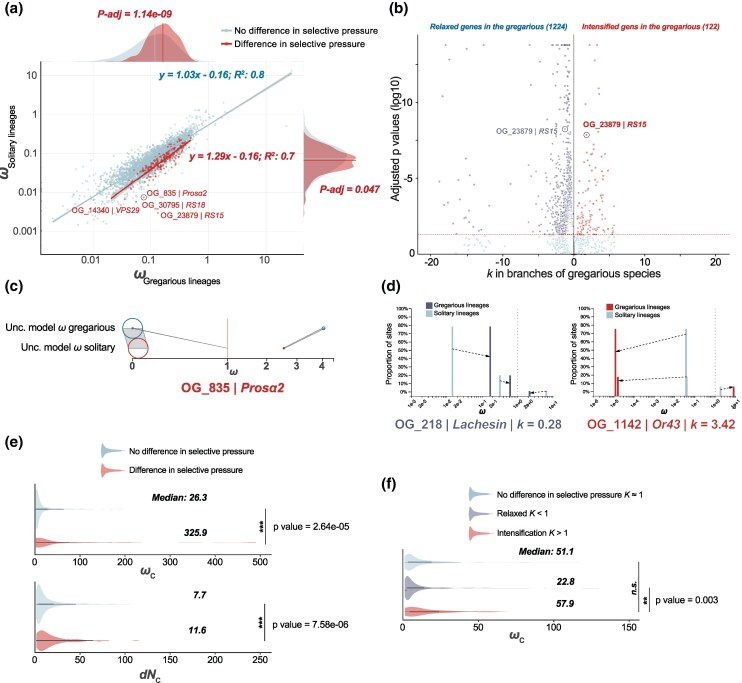
Molecular evolution in scOGs and their association with signature convergent molecular evolution in gregarious lineages. a) Scatter plot of scOGs showing differential selecting regimes between gregarious and solitary lineages. Red dots correspond to genes that are differentially selected in gregarious lineages compared with solitary lineages, while light blue dots are not. Regression lines show significant slopes. Along *x* and *y* axes, the distributions of *ω* in gregarious and solitary lineages for the genes associated with gregarious and solitary lineages are plotted. b) Scatter plot of Relax analysis. Light-purple dots represent genes under relaxation (*k* < 1; *P*_adj_ < 0.05), while red dots correspond to genes under intensification (*k* > 1; *P*_adj_ < 0.05). *x* axis is log_2_-transformed to center *k* = 1 to 0. The red-dashed line corresponds to the *P*_adj_ threshold of 0.05. c) Plot showing the three-rate classes of *ω* between gregarious versus solitary lineages in *Prosα2*, as an example of Busted-ph test result. d) Histograms showing the three-rate classes of *ω* between gregarious versus solitary lineages in *Lanchesin* and Or43. The two are examples how rates changes due to a relaxed (left) and intensified selection (right), in the Relax test. e) Violin plots showing the rate of convergent positive selection (*ω_C_*) and nonsynonymous convergent substitutions rate (*dN_C_*) between relaxed (light purple) and intensified (red) genes. f) Violin plots showing the distribution of *ω_C_* between not-differentially selected (light blue), relaxed (light purple), and intensified (red) genes. In all plots asterisks the size of the *P*-value: *n.s.* = not significant; *<0.05; **<0.01; ***<0.001.

Our Busted-ph (Kosakovsky Pond et al. 2020; Pond 2023) analysis detected 203 scOGs (6%) to be under distinct selection regimes in gregarious lineages (adjusted *P* (*P*_adj_) < 0.05; Fig. 2a and e; supplementary table S1, Supplementary Material online). While the median strength of selection acting on scOGs in solitary (*ω_s_*) and gregarious lineages (*ω_g_*) is only marginally different overall (0.072 for not differentially selected genes versus 0.066 for DSGs; one-sided Wilcoxon rank-sum test *P*_adj_ = 0.047), we detect a much higher median *ω_g_* for those genes with distinct selection regimes between solitary and gregarious lineages (0.12 vs. 0.16; one-sided Wilcoxon rank-sum test *P*_adj_ 2.2 × 10^−16^; Fig. 2a). This is associated with a proportion of nucleotides evolving under strong purifying selection in the solitary lineages, which shift toward neutrality or positive selection in gregarious lineages (Fig. 2c). Our Relax analysis (Fig. 2b) supports a role for increased neutrality in a class of scOGs in gregarious lineages, with 36% (1,224 scOGs) showing a signature of relaxation (supplementary table S1, Supplementary Material online). Among this class of genes, no GO terms are significantly enriched but we note potential candidates with functions similar to those highlighted elsewhere. For example, several genes are associated with the response to viruses, including a *Lachesin*-homolog gene (Fig. 2d) and other ubiquitin-related genes (e.g. *sina*, an E3 ubiquitin-protein ligase). In other insects, viruses have been demonstrated to interact with *lachesin* proteins during their attachment to neurons (Rana et al. 2020), and ubiquitination is known to play an important role in viral infection (Chaudhary et al. 2023). In *Bombyx mori*, a *sina*-homolog is downregulated to inhibit cellular interactions with viral proteins (e.g. *BmNPV*) (Wang et al. 2021). A smaller proportion of scOGs also show evidence of intensified positive selection in gregarious lineages (∼4%, 122 scOGs; *P*_adj_< 0.05; Fig. 2b; supplementary table S1, Supplementary Material online). Among these genes, an odorant receptor (OR43; ortholog of the *B. mori* OR49; Cicconardi et al. 2024) (Fig. 2d) is known to bind to the antimicrobial and larval food attractant compound citral (Hamamura and Naito 1961; Carraher et al. 2012).

**Fig. 3.**
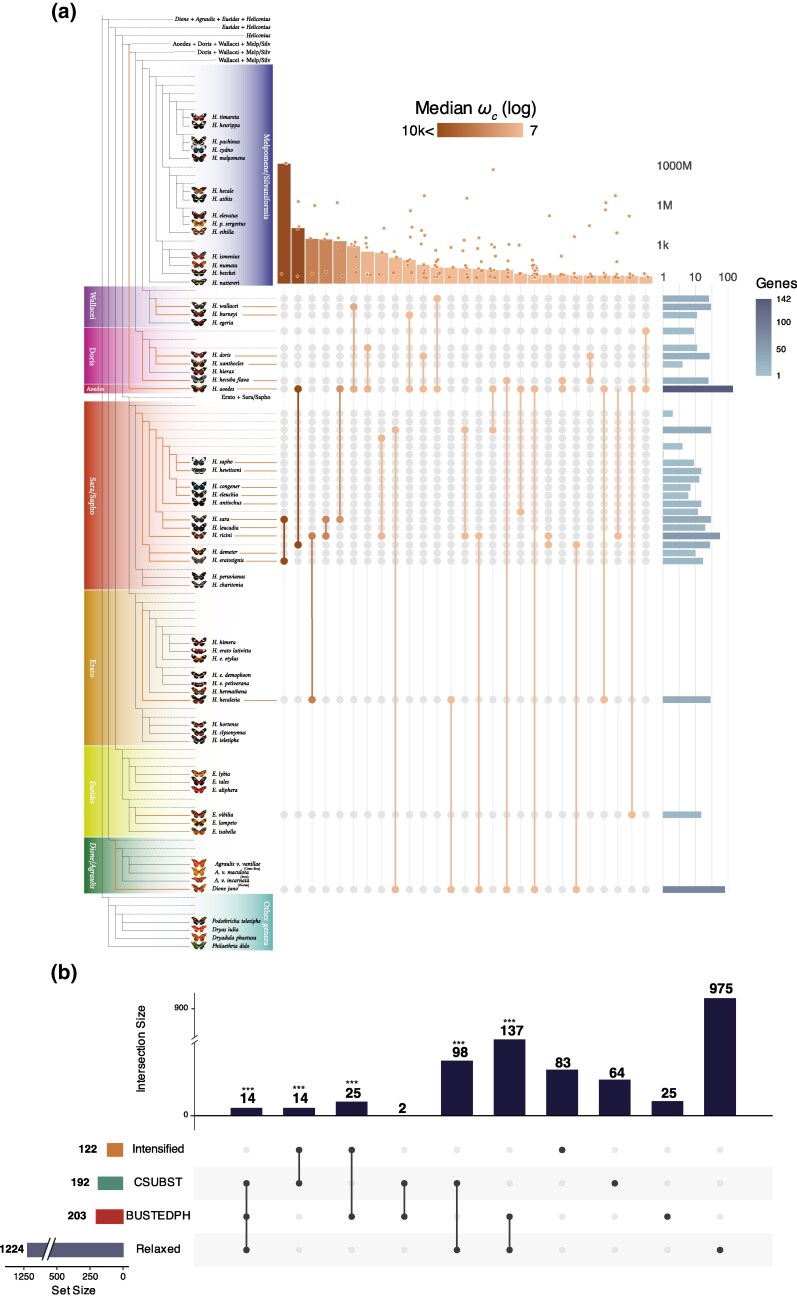
Convergence of molecular evolution in scOGs in gregarious lineages. a) Csubst analysis across Heliconiini phylogeny. Next to the expanded tree diagram shows the lineages involved in convergent positive selection (*ω^+^_C_*). The histogram on top shows the median value of *ω^+^_C_* for specific pairs. On the right-most side the histogram shows the number of genes with evidence of convergent evolution involving a specific branch. b) UpSet plot showing the intersection of the four gene lists. On top of the vertical histogram the corresponding *P*-values of the probability of the overlap being due to a random distribution. Asterisks indicate *P*-values: ***<0.001.

Our Csubst (Fukushima and Pollock 2023) analysis next sought to identify convergent substitutions in gregarious lineages. We first assessed the degree of convergent selection in genes previously identified as evolving under differential selection (Busted-ph), relaxation, or intensification (Relax). To do so, we selected a relatively low threshold for convergence of selection (*ω_C_* > 1.0) and rate of nonsynonymous convergence (*dN_C_* > 1.0). Genes under differential selection showed a strong increase in both *ω_C_* and the rate of nonsynonymous convergent substitutions (medians: 325.9 *ω_C_* and 11.6 *dN_C_*) compared with other scOGs, where no differential selection was detected (medians: 26.3 *ω_C_* and 7.7 *dN_C_*; one-sided Wilcoxon rank-sum test *P*_adj_ < 2.7 × 10^−5^) (Fig. 2e), with no significant difference in the rate of synonymous convergent substitutions (*dS_C_*), which can function as a negative control (Fig. 2f). For scOGs under relaxed and intensified selection, there is less enrichment of for signals of convergence, but in gregarious lineages, scOGs under intensified positive selection show a stronger enrichment (higher values on average) of *ω_C_* compared with scOGs under relaxed selection (*ω_C_* medians: 57.9 vs. 22.8; one-sided Wilcoxon rank-sum test *P*_adj_ < 3.0 × 10^−3^). This pattern suggests that the intensified positive selection in gregarious lineages is partly driven by convergent substitutions. Finally, selecting a more stringent threshold of *ω_C_* > 5.0 and *dN_C_* > 2.0 for an *arity* ≥ 2, we were able to identify 192 scOGs under convergent positive selection (*ω_C_*^+^) (Fig. 3a and b; supplementary table S2, Supplementary Material online). Most of this convergence occurred between pairs of gregarious lineages (*arity* = 2) (Fig. 3a). The most frequent lineages under *ω_C_*^+^ were *Heliconius aoede* and *D. juno* (32 scOGs with median *ω_C_* of 9.9), which split from their MRCA more than 25 mya (Fig. 1). Among these genes, we find *Herc4*, a ubiquitin ligase4, *Notch*, and *Neurotrophin 1*, which are all connected with Toll-related receptors involved in host defense (Zhang et al. 2012), and a (cytosine-5) tRNA methyltransferase (*Mt2*), which in *Drosophila* is implicated in the innate immune response and lipid homeostasis (Abhyankar et al. 2018). *Heliconius aoede* also has the highest number of scOGs under *ω_C_*^+^, with over 100 genes, followed by *D. juno* and *Heliconius ricini* (Fig. 3a). The scOGs implicated in convergence among the highest number of lineages are a *Rcc1*-homolog and *kazachoc* (*kcc*). *Rcc1* has roles in cell proliferation, cell survival, apoptosis, epigenetic regulation, and neuronal specification (Dasso et al. 1992; Hadjebi et al. 2008). *Kazachoc* encodes a potassium:chloride symporter expressed in the insect central nervous system and is implicated in modulating neural firing rates (Chen and Swale 2023). We note that the majority of our signals of convergent positive selection are detected among subsets of lineages that evolved gregarious larvae, rather than across all lineages. We interpret this as an indication that convergent selection does not act precisely on the same sites, and the same genes across the phylogeny, but reflects an intricate mosaic of selection acting across multiple molecular pathways, within which some loci are prone to repeated selection.

Exploring overlap between genes highlighted by the independent analyses above, we find significantly more overlap than expected by chance in four of the six pairwise datasets (100,000 permutations; *P* < 0.008; Fig. 3b). The most interesting overlap between genes under relaxation, differentially selected, and under *ω_C_*^+^ includes 15 scOGs. Among them are *CycB3*, *Glg1*, *Dsp1*, and *Fancl*, which are implicated in the regulation of cell cycle, Golgi apparatus, and response to DNA damage stimuli (Reggiori et al. 2003; Hodson et al. 2011; Planche et al. 2012; Garrido et al. 2020), and *norpA*, which in *Drosophila* plays a prominent role in the transduction of light and environmental stimuli (Bloomquist et al. 1988). Overall, our analysis of PCGs suggests a shift toward gregariousness affects the selection regimes shaping multiple biological processes, with a strong signal of convergence among a small subset of genes.

### A Subset of CNEEs Show Accelerated Rates of Evolution in Gregarious Lineages

**Fig. 4.**
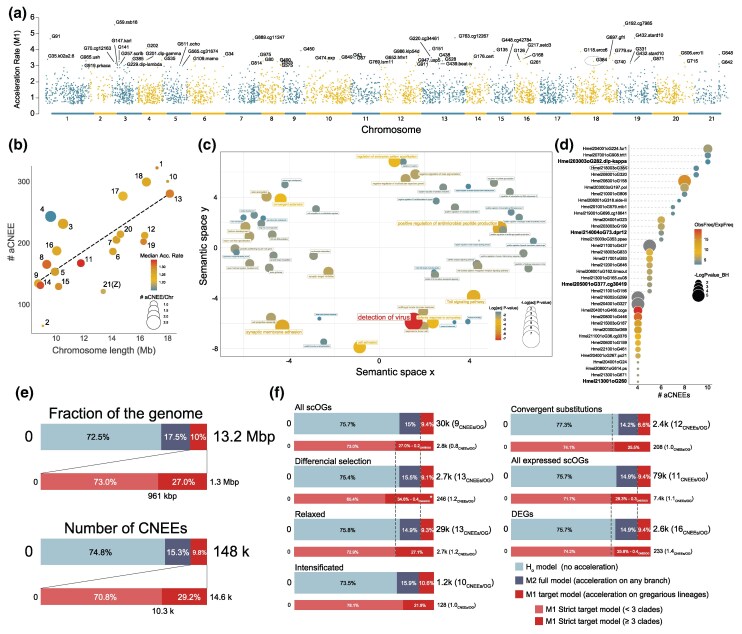
Convergent evolution of conserved non-coding elements in gregarious lineages throughout Heliconiini phylogeny. a) Distribution of “strict” aCNEEs across the genome of *H. melpomene*, used as reference. *y* axis is the acceleration rate under the M1 model. b) Scatter plot showing a positive correlation between the number of aCNEEs and chromosome lengths. Redshift shows an enrichment of high rate. In particular, chromosomes 11 and 14 show on average higher rates, while chromosome 4 shows an excess of aCNEEs with respect to its size, but with low rate on average, while chromosome 21, the sex chromosome (Z), and 2 show a very low number of aCNEEs. c) Semantic plot of the enriched GO terms using genes interested by *strict* aCNEEs present in putative regulatory elements (see Materials and Methods). Large and redshift circles correspond to GO terms that have smaller *P*-values. d) Bubble plot of some of the 73 genes enriched with aCNEEs in their regulatory elements (supplementary table S4, Supplementary Material online). Circle size shows the log_10_ of the *P*_adj_ values of the enrichment, while the redshift shows higher values of the ratio between the observed versus the expected frequency. Genes that are also differentially expressed in at least one comparison are listed in bold. e) Stacked barplots showing the proportion of the CNEEs as fraction of the genome (top) and in terms of model distribution (bottom). f) Stacked barplots showing the proportion of the different classes of CNEEs in different gene sets, including expressed and differentially expressed scOGs (DEGs). The asterisk indicate *P* < 0.05, *n.s.* = not significant.

To determine the extent of convergent molecular evolution at noncoding loci, we next compiled a total of 148k CNEEs across the 63-way genome alignment (Cicconardi et al. 2023), using *H. melpomene* as a reference. CNEEs are small genomic regions previously shown to be enriched with *cis*-regulatory elements (Cicconardi et al. 2023; Xue et al. 2023). Within these 148k CNEEs, we explored convergent shifts in molecular rate associated with transitions to gregarious behavior (Sackton et al. 2019) (Fig. 4). We identified 15.3% of CNEESs that show variable rates of evolution across the phylogeny (acceleration under a “full model” M2 has a higher probability, expressed as Bayes Factor cutoff 1.0, than “null model” M0) and 9.8% that show convergent acceleration (aCNEEs) in gregarious lineages (acceleration under a “target model” M1 has a higher probability than M0 and M2), corresponding to ∼10% (1.32 Mb, 14.6k CNEEs) of the reference genome. Of these regions, roughly a third (29.2%) of all aCNEEs are convergently accelerated in at least three different clades of the phylogeny (Fig. 4a and b).

Taking all aCNEEs together, they are enriched in putative regulatory domains of genes associated with more than a dozen GO term, depending on the different tests and FDR corrections applied (Fig. 4c; supplementary table S3, Supplementary Material online). The most significantly enriched GO terms include those associated with viral detection, defense response to oomycetes and fungi, regulation of antimicrobial peptide production, and Toll signaling pathway, the innate immune response pathway in insects. We found 37 candidate genes associated with gregarious behavior that harbor more *strict* aCNEEs than expected by chance (Fig. 4a and d; supplementary table S4, Supplementary Material online; *P*_adj_ < 0.05). These include the putative *phenylalanyl-tRNA synthetase β-subunit homolog* (*β*-PheRS). *β*-PheRS is an enzyme that acts by charging tRNAs with their cognate amino acid and, in *Drosophila*, manipulating *β*-PheRS expression affects growth speed, the timing of pupation, and behaviors including feeding and roaming (Brunßen and Suter 2024).

Finally, we explored the intersection between CNEEs and scOGs and found that in putative regulatory domains of the scOGs, 15% of the CNEEs are accelerated (M2) and 9.4% are convergently accelerated (M1), of which 27% are accelerated in at least three clades (Fig. 4e). These are similar numbers to our genome-wide analyses, suggesting that our scOG dataset is a robust representation of the entire coding gene repertoire. Focusing on scOGs with putative associations with gregarious behavior (i.e. differentially selected, relaxed, intensified, and positively converging genes), we found a significant enrichment of strict aCNEEs (*P* < 0.05; 34.6% of CNEEs) in the putative regulatory domains of scOGs with differentially selective regimes in gregarious lineages. This translates to a 2-fold greater density of *strict* aCNEEs in the differentially selected scOGs (0.4 aCNEE/scOG) compared with the background scOGs (0.2 aCNEE/scOG). Although not statistically significant, we also observed that aCNEEs in putative regulatory domains of differentially selected scOGs seem to have slightly higher acceleration rates (median: 1.31 vs. 1.28) and lower conservation rates (median: 0.091 vs. 0.093) (supplementary fig. S2, Supplementary Material online). Overall, our analyses of noncoding genomic regions show a strong signal of convergence associated with gregarious larvae. Importantly, the combination of analytical approaches applied to both coding and noncoding regions allows us to detect that selection for gregarious behavior is simultaneously acting at the same loci: in protein-coding regions, by changing amino-acid composition (purifying and/or positive selection), and through accelerated rates of sequence evolution in *cis*-regulatory elements (noncoding elements).

### Shifts in Gene Expression During the Independent Evolution of Gregarious Behavior

**Fig. 5.**
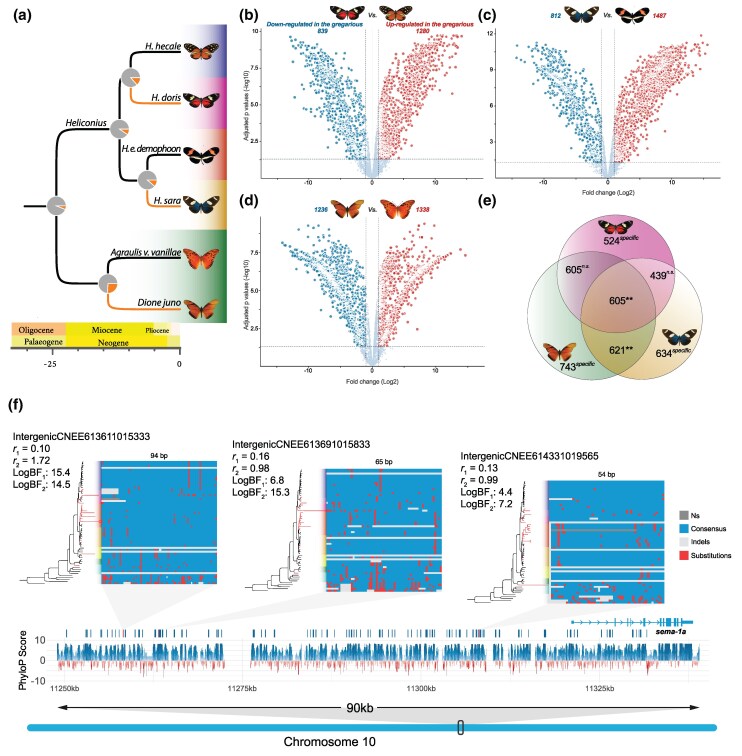
Convergent gene expression analysis across three pairs of species across the Heliconiini phylogeny. a) Dated phylogeny of the three species pairs used as proxy to study the gene expression convergence in gregarious lineages. As in Fig. 1, orange branches indicate lineages with gregarious larvae, while pie charts indicate the ancestral state reconstruction of the most recent common ancestor having gregarious larvae. b to d) Volcano plot of the differentially expressed scOGs in the three pairs of species. In each pair, the solitary larva species was used as “control”; therefore, red points correspond to upregulated genes in the gregarious species, while the light-blue points represent downregulated genes. Horizontal dashed lines indicate *P* = 0.05; vertical dashed lines indicate a ± 1 log_2_FC threshold. e) Venn diagram of the DEGs in common in the three studied pairs. Asterisks correspond to the size of the *P*-value: *n.s.* = not significant; **<0.01. f) Genomic locus around the gene *sema-1a*, differentially expressed in all three pairwise comparisons. The locus has a number of conserved loci (blue) both in to close the TSS, and more distal proximity. Below them PhyloP CONACC tracks showing log-transformed *P*-values of conserved (blue) and accelerated (red) nucleotides. The locus has three aCNEEs (red) for each of them a phylogenetic tree is shown with branch lengths proportional to the corresponding acceleration rates for the gregarious lineages (red branches). Next to the phylogenetic tree, the alignment of the CNEE is also shown. For each aCNEE, the conservation (*r_1_*), acceleration rates (*r_2_*), and their respective log of the Bayes factors (logBF) are also listed.

To further explore the impact of convergent shifts toward gregarious behavior on neural gene expression, we collected larval brain transcriptome data for three phylogenetically independent pairs of solitary/gregarious species (Fig. 5a), with five replicates per species. Data normalization, aligning gene lists between species pairs, and removing genes that had zero reads across all species resulted in a final dataset of 4,874 scOGs shared across all six species (obtained from Cicconardi et al. 2023). We next compared patterns of gene expression between each pair of solitary/gregarious species. This revealed a relatively high number of scOGs with significant differential expression within each pair (Fig. 5b to d; supplementary table S5, Supplementary Material online), with an apparent trend toward fewer DEGs between pairs that are more recently diverged (Fig. 5a). Separate tests for functional enrichment on DEGs between species pairs revealed no significantly enriched GO terms after Benjamini–Hochberg *P*-value correction for false detection rate (all *P* > 0.05; supplementary table S6, Supplementary Material online).

We next examined the amount of DEG overlap between pairs. Permutation tests revealed that the level of DEG (upregulated and downregulated in gregarious lineages) overlap between the DEG sets derived from the separate *D. juno* versus *A. v. vanillae* and *H. doris* versus *H. hecale* comparisons is significantly greater than expected by chance (overlapping DEGs comprised 47% of the total DEGs in the *D. juno* vs. *A. vanillae* set and 56% to total DEGs from the *H. doris* vs. *H. hecale* set; *P* < 0.001; Fig. 4e; supplementary table S7, Supplementary Material online). The degree of DEG overlap at the intersect between all three pairwise comparisons is also significantly greater than expected by chance (23%, 28%, and 26% in *D. juno* vs. *A. v. vanillae*, *H. doris* vs. *H. hecale*, and *H. sara* vs. *H. erato sets*, respectively; *P* < 0.001; Fig. 5e; supplementary table S7, Supplementary Material online). The higher strength of overlap between *D. juno* and *H. doris* is notable, as the larvae of these species are more strictly gregarious throughout larval development, relative to *H. sara* (Muyshondt and Young 1973; Jiggins 2017). Considering overlapping upregulated and downregulated genes separately, the level of upregulated gene overlap between *D. juno* and *H. doris* is again significantly greater than expected by chance (29% and 30%, respectively; *P* = 0.002; supplementary table S7, Supplementary Material online), and the same is true for the upregulated genes at the intersect between all three pairs (10%, 10%, and 9% in *D. juno*, *H. doris*, and *H. sara*, respectively; *P* = 0.008). In contrast, overlap among downregulated genes does not deviate from random expectation (supplementary table S7, Supplementary Material online).

We next used the transcriptomic data to evaluate how expression evolved along the scOGs using the dated phylogenetic tree, employing the phylogenetic multioptima OU models (Hansen 1997), to allow for possible adaptive shifts of optimal expression levels. This modeling approach can identify statistically supported expression regime shifts (Hansen 1997; Khabbazian et al. 2016) on each scOG. The model has two parameters: a rate of adaptation (*α*) and a rate of stochastic evolution (*σ²*). The first quantifies how strongly a trait is pulled toward an optimal value. When this rate approaches 0, then the shift is approaching the Brownian motion (no adaptation), with high values reflecting strong selection. The rate of stochastic evolution, instead, represents the variance of the Brownian motion component, which means that low values are associated with more stable trait evolution, while high values to random fluctuation. Although the OU analysis has most suitably been applied to large phylogenies (≥50 species) (Duttke et al. 2024), we cautiously applied it knowing that our framework may have very low power (supplementary table S8, Supplementary Material online). We detected convergent evolution in 185 scOGs, of which 162 (89%) were also found to be differentially expressed in the pairwise analysis. The model detected strong or very strong selection (*α* > 1) and moderate or very low variability (*σ*² > 1), indicating a fast convergence in a large proportion (47%) of the scOG highlighted here, while rapid adaptation with noise (*α* > 1; *σ*² < 1) was detected in a further 23%, with the remaining 30% displaying a signal of drift (*α* < 1; *σ*² > 1).

Considering scOGs with at least three aCNEEs in their putative regulatory regions, we identified 14 of them that are differentially expressed between one or more pairs of solitary and gregarious Heliconiini (supplementary table S3, Supplementary Material online). Six of which harbor more aCNEEs than expected by chance (supplementary table S3, Supplementary Material online; *P*_adj_ < 0.05). Of particular note is *dpr12* (Fig. 4d), which is differentially expressed in all three species pairs, and is involved in dopamine signaling, a neuromodulator with known regulatory roles in several insect behaviors linked to sociality, including feeding behavior and aggression (reviewed in Verlinden 2018), and two *semaphorins* (*sema-1a and sema-1b*, the former is shown in Fig. 5f), which is involved in growth cone guidance through its role in axonal repulsion (Nguyen et al. 2022).

Several of the convergent DEGs, those overlapping at the intersect between at least two species-pair comparisons, with the greatest mean log_2_FC identified in our study are predicted to be involved in functions putatively linked to social behavior, such as hormonal pathways (*5htr*, *dop2r*), sensory system development and function (*CG31559*, *ap-1σ*, *hdc*, *dan*), immune response (*rnf19b*, *CG3829*, *samdc*, *senju*, *ubc7*), feeding behavior and metabolism (*CG3552*, *mthl3*, *rmi1*, *hcrtr2*, *CG30344*, *indy*), and neural development (*tao*) (supplementary table S7, Supplementary Material online). While genes associated with sensory functions may provide clues as to the stimuli detected by social larvae, the convergent upregulation of genes controlling hormonal pathways, such as serotonin and dopamine receptors, may also suggest that modulation of how social cues are used plays an important role in the evolutionary transition to social behavior. As observed in other species, the activity and regulation of these hormones can be crucial in the production of the social behavioral phenotype (Anstey et al. 2009; Vleugels et al. 2015; Sasaki et al. 2021), potentially because of their involvement in feeding (Dacks et al. 2003; Neckameyer 2010; French et al. 2014) and aggression or attraction/repulsion responses between conspecifics (Dierick and Greenspan 2007; Anstey et al. 2009; Sasaki et al. 2021). Elsewhere, these candidate genes may be associated with secondary selection pressures that result from social living. For example, feeding regulation is likely to be an important social phenotype given higher competition for resources between group members (Nilsson and Forsman 2003; Reader and Hochuli 2003) and the greater risk of starvation during collective defoliation of a host plant (Denno and Benrey 1997; Daly et al. 2012). Similarly, social organisms are more susceptible to disease, given their close proximity to one another and sharing of food sources (López-Uribe et al. 2016), and group living is hypothesized to be associated with increased selection on individual immunity (Evans et al. 2006; López-Uribe et al. 2016). Given the lack of studies on the genetic basis of social behavior outside of eusocial insects (Hymenoptera/Blattoidea), it is difficult to determine if our results highlight more general pathways involved in social traits across insects. Nonetheless, there is an interesting overlap with previous work that identified a shared genetic toolkit for caste determination among social transitions in vespid wasps, which also highlighted the Toll pathway, ubiquitination, ion transport, and locomotion behavior (Wyatt et al. 2023; Catto et al. 2024).

Finally, to further explore the putative regulatory functions of the aCNEEs, we took the strict aCNEEs that overlap with the putative regulatory regions of scOGs that also show differential selection between gregarious and solitary lineages. We then performed a test of whether they show any signal of enriched motifs, using all strict aCNEEs overlapping with all tested scOGs as a null distribution. The analysis identified 48 enriched motifs with a relative high fold enrichment, which range from ∼2-fold up to ∼13-fold (supplementary table S9, Supplementary Material online). Within the 20 most enriched motifs are those associated with transcription factors that are regulators in embryonic patterning (bcd, zen, cad, tll, vnd), organogenesis (tin, vvl, Ets98B), or sensory/neural specification and neurogenesis (vnd, sens, dsf, TfAP-2, vvl, Ets98B). Others like pho, Dref, and bigmax function at a chromatin or proliferation, influencing transcriptional readiness or metabolic activity.

## Conclusions

Using multiple approaches across transcriptomic, protein-coding, and noncoding genomic data, our analyses provide a robust case that convergent transitions toward gregarious larval behavior are associated with genome-wide convergent molecular evolution. We have identified PCGs under differential selection in gregarious lineages, suggesting that changes at these loci may be important in the evolution of the gregarious phenotype. In particular, we found a noticeably high number of genes under relaxation associated with the shift to the gregarious behavior. A similar pattern was also found in the social parasitic wasp *Vespula squamosa* where it was found a 2-fold ratio of relaxed versus intensified genes, suggesting that a behavioral change can have a dramatic effect on selective pressure (López-Uribe et al. 2016). Another example can be found in spiders where the transition to sociality is associated with elevated *ω* almost entirely due to relaxed selection (Tong et al. 2022; Ma et al. 2025). We also reveal a parallel signal of convergence at putative *cis*-regulatory elements, providing evidence of common shifts in selection affecting coding and noncoding elements of a wider locus. Although signatures of introgression have been found between many lineages within the Heliconiini tribe, there is no evidence of introgression between lineages that have independently evolved gregarious larvae (see analyses in Cicconardi et al. 2023 and Fig. 2 and supplementary fig. S25, Supplementary Material online therein). This is also evident in the aCNEEs shown in Fig. 5f, where each lineage shows a different sequence pattern. Finally, we have taken the first steps in exploring the gene expression profiles of gregarious species in relation to solitary species, revealing a convergent pattern of differential expression in association with convergence in the gregarious phenotype, with a number of genes associated with convergent aCNEEs being differentially expressed in independent gregarious lineages.

We note that a formal quantification of gregarious behavior, including the potential differences between “levels” of gregariousness, is lacking, with only the clutch size range available as a potential metric of group size within Heliconiini (Brown 1981; McLellan and Montgomery 2024). Of course, behavioral differences between gregarious species, such as those relating to how individuals organize and interact with one another, could be associated with considerable differences in the genome and transcriptome, which we will not have accounted for in this study. One future possibility, for example, will be to use continuous metrics of gregariousness, which can be implemented with PhyloAcc-GT and Phylog2p (Macdonald et al. 2025), which may result in refined signals of molecular evolution. However, such an analysis would require substantial effort in improved phenotyping across many species. Nevertheless, our comprehensive analyses identify the first candidate loci associated with gregarious larval behavior in Lepidoptera, in particular highlighting the serotonergic and dopaminergic pathways as a potential mechanism for transitioning to larval gregariousness. Given the importance of these molecular mechanisms on other insect social behaviors, our data provide candidate loci for future functional investigation. Crucially, our data also reveal the likely importance of genes involved in a wide range of physiological and behavioral traits, which likely evolved in concert with gregariousness per se. These include feeding behavior, immune system function, and possibly aggression, as components of the repeated evolution of the gregarious phenotype across the Heliconiini. Little is known of the specific “tolerance” genes that are likely to facilitate social behaviors (Mayhew and van Alphen 1999), and our data provide a first step toward the identification of genes involved in anti-aggression, anti-cannibalism, and the prevention of egg consumption. More broadly, our analyses highlight the rich potential of phenotypically informed comparative analyses of densely sampled genomic datasets of closely related, but phenotypically divergent, phylogenetic groups.

## Supplementary Material

msaf179_Supplementary_Data

## Data Availability

The code used for this study and statistical analysis are available in the Git repository: https://github.com/francicco/-ConvergentMolEvolutionGregLarvalBehaviour.
