# Supplementary material for: Convergent Molecular Evolution Associated With Repeated Transitions to Gregarious Larval Behavior in Heliconiini
Source: Mol Biol Evol. 2025 Jul 29;42(8):msaf179. doi: 10.1093/molbev/msaf179 (PMC12342998; doi:10.1093/molbev/msaf179)
Supplement: msaf179_Supplementary_Data [file msaf179_supplementary_data.zip › SocialCats_SupplementaryFigures_Final (1).pdf]

Supplementary Figures of: *Convergent molecular evolution associated with repeated transitions to gregarious larval behaviour in Heliconiini*

Authors: Francesco Cicconardi[1*], Callum F. McLellan[1*], Alice Seguret[1], W. Owen McMillan[2], Stephen H. Montgomery[1,2]

* *contributed equally*

Affiliations:

[1] School of Biological Sciences, University of Bristol, Bristol, United Kingdom;

[2] Smithsonian Tropical Research Institute, Gamboa, Panama

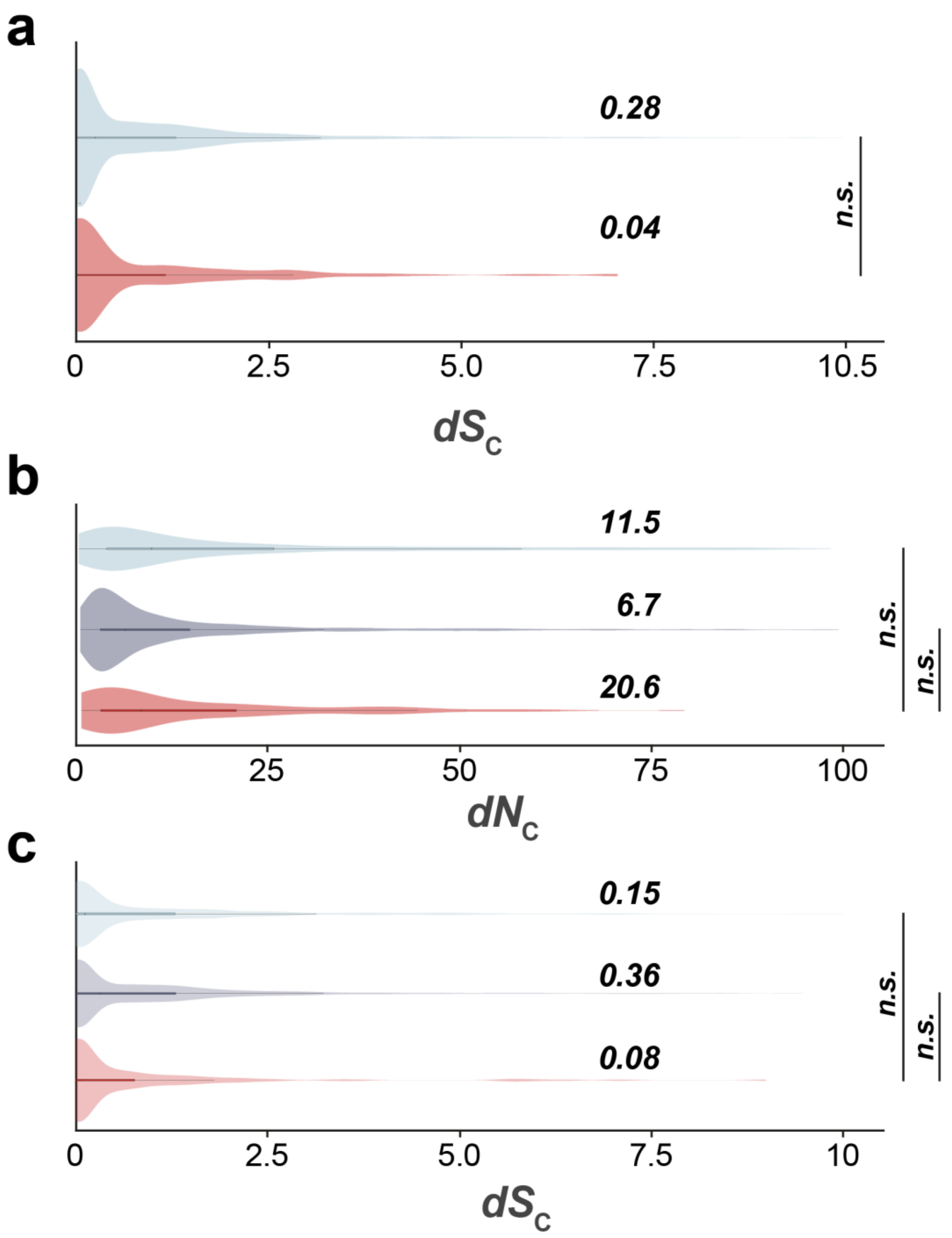

Supplementary Figure 1. Exploration of convergent molecular evolution in BUSTED-PH and RELAX in association with gregarious lineages. (a) Violin plots showing the rate of synonymous convergent substitutions (dS_C) in genes that are not differentially selected (light blue) and genes that are differentially selected between solitary/gregarious lineages (red). (b) Violin plots showing the rate of non-synonymous convergent substitutions (dN_C), and the (c) rate of synonymous convergent substitutions (dS_C) between genes that show no difference in selection regime (light blue), or that show relaxed (light purple), or intensified (red) selection in gregarious lineages genes. In all these tests no significant difference between groups was identified.

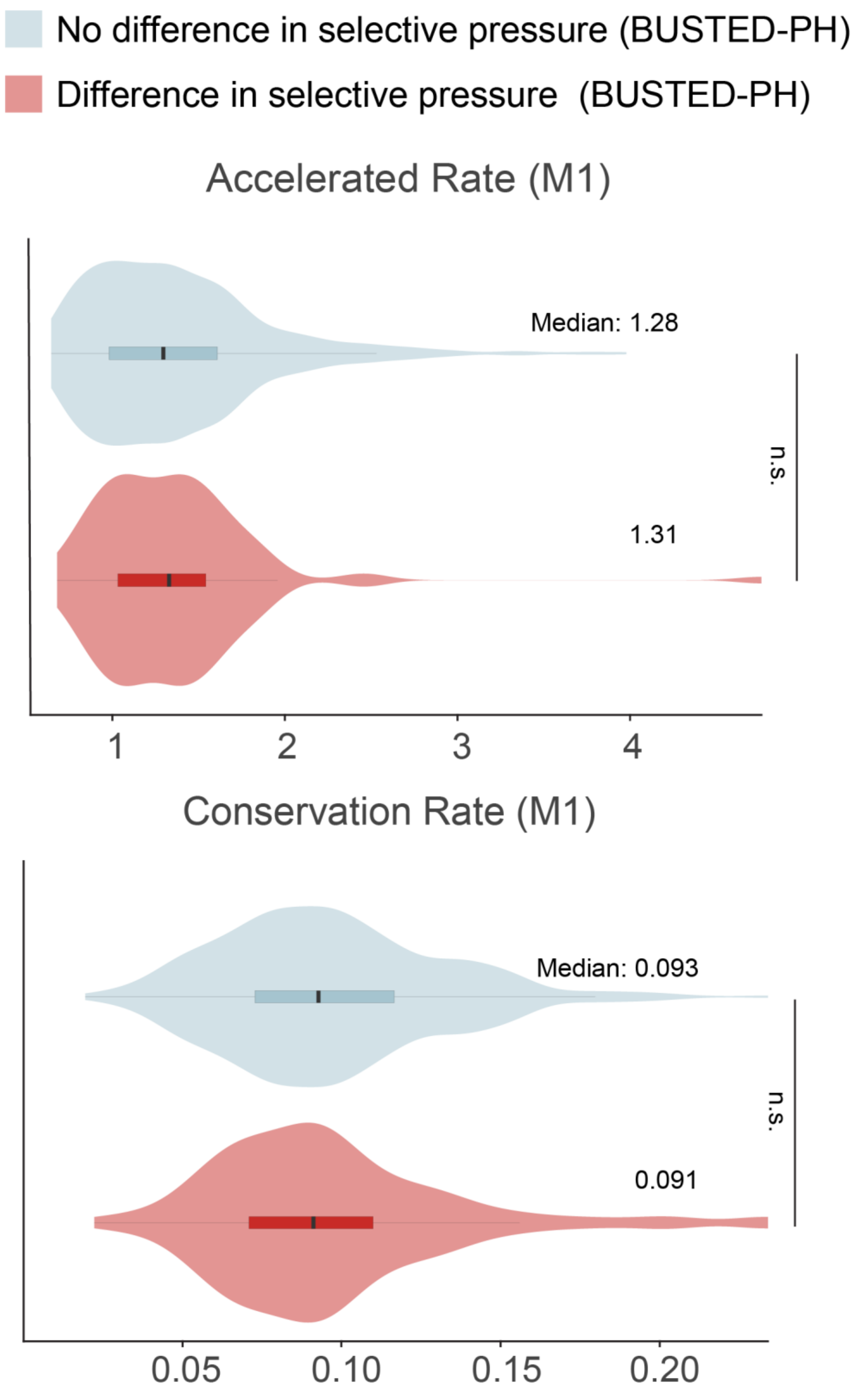

Supplementary Figure 2. Conservation and acceleration rates associated with BUSTED-PH tested genes. Violin plots showing the acceleration (top) and conservation (bottom) rates of CNEEs in association with non-differentially (light blue) and differentially (red) selected genes between gregarious/solitary lineages (BUSTED-PH gene set).
